# Differences in brain functional connectivity between autonomous sensory meridian response and classical music

**DOI:** 10.3389/fnins.2026.1815703

**Published:** 2026-06-04

**Authors:** Misuzu Oishi, Kei Sasaki, Noriko Sakurai, Naoki Kodama

**Affiliations:** 1 Laboratory Department of Radiological Technology, Niigata University of Health and Welfare, Niigata, Japan; 2 Department of Radiological Science, Gunma Paz University, Takasaki, Japan

**Keywords:** autonomous sensory meridian response, default mode network, fMRI, functional connectivity, salience network

## Abstract

Autonomous sensory meridian response (ASMR) is a tingling sensation that originates in the occipital region and spreads along the neck and spine, elicited by specific audiovisual stimuli known as ASMR triggers. The characteristics of ASMR-related changes in brain activity relative to other external stimuli, and whether these changes are specific to ASMR, remain unclear. The aim of this study is to compare changes in functional connectivity during exposure to ASMR triggers and classical music, and to clarify the changes in connectivity that are more strongly associated with ASMR trigger listening. Forty-eight healthy adults without a history of psychiatric disorders underwent functional MRI under three conditions: resting state without auditory stimulation, listening to ASMR triggers, and listening to classical music. Functional connectivity during the ASMR and classical-music conditions was assessed relative to the resting state. As a result, functional connectivity between the medial prefrontal cortex and the right lateral parietal cortex increased during ASMR trigger listening compared to rest. Relative to classical music listening, ASMR trigger listening increased functional connectivity between the medial prefrontal cortex and the right lateral parietal cortex, between the left anterior insula and left supramarginal gyrus, and between the right rostral prefrontal cortex and the anterior cingulate cortex. No significant changes in functional connectivity were observed during classical music listening alone. These findings suggest that, compared with classical music listening, ASMR trigger listening is associated with stronger functional connectivity between specific ROI pairs involved in self-referential/internal evaluative processing and sensory integration.

## 1 Introduction

Autonomous sensory meridian response (ASMR) is a tingling sensation that begins in the occipital region and extends down the back, elicited by specific auditory or visual stimuli known as ASMR “triggers” (Barratt and Davis, 2015). Beyond its sensory qualities, ASMR has been associated with reductions in depressive symptoms and psychological stress, suggesting its potential use as a relaxation modality (Barratt and Davis, 2015). Physiological studies have shown concurrent decreases in heart rate and increases in skin conductance during ASMR, suggesting a complex autonomic profile in which both sympathetic and parasympathetic systems are engaged (Poerio et al., 2018). Neuroimaging research using task-based functional magnetic resonance imaging (fMRI) in which participants watched videos containing ASMR triggers identified activation in cortical regions implicated in cognition, including the prefrontal and cingulate cortices (Smith et al., 2019). In ASMR-sensitive individuals, additional activation has been observed in the nucleus accumbens, part of the reward-processing system, and in the insular cortex, which is involved in emotion generation (Lochte et al., 2018). Despite these insights, the broader characteristics of ASMR-related brain activity relative to other external stimuli, and whether these changes are specific to ASMR, remain unclear. Direct comparisons with non-ASMR stimuli may therefore help clarify whether the observed neural changes are specific to ASMR.

Classical music provides a useful comparison, as it can evoke strong positive affective responses (Blood and Zatorre, 2001). Task-based fMRI studies involving classical music have reported activation in the anterior cingulate cortex, as well as the hippocampus and amygdala, which are involved in emotion processing and memory (Mitterschiffthaler et al., 2007). In connection with this type of brain activity, listening to classical music triggers the recall of autobiographical memories and a sense of self-absorption (Taruffi et al., 2017). A direct comparison of ASMR trigger sounds and classical music revealed overlapping but distinct activation patterns, in which ASMR triggers elicited stronger activity in the superior frontal and lingual gyri, as well as selective activation of the insula and nucleus accumbens (Sakurai et al., 2021). Thus, while ASMR and classical music activated some overlapping brain regions, many regions were unique. These observations indicate that while both ASMR and music engage emotion-related circuits, they may rely on partially distinct mechanisms of cognitive and affective processing.

Furthermore, neural information processing for such stimuli is supported not only by isolated regions alone but by the dynamic interactions of large-scale brain networks (Song et al., 2023). Consequently, network-level evaluation is required to investigate the neural basis of ASMR. Functional connectivity analyses, which quantify synchronous fluctuations in blood-oxygen-level-dependent (BOLD) signal between brain regions, have been used to assess the cooperative activity of brain networks and provide a framework for evaluating how sensory stimuli influence brain-wide information processing (Li et al., 2022). Among the networks most relevant to sensory and emotional processing are the default mode network (DMN), frontoparietal network (FPN), and salience network (SN). The DMN is most active at rest and is involved in internally directed mentation such as self-referential processing (Buckner et al., 2008). The FPN is recruited during cognitively demanding tasks and supports goal-directed decision-making by maintaining and manipulating information in working memory (Menon, 2011). The SN mediates the switching between the DMN and FPN and promotes appropriate responses by selecting the most behaviorally relevant stimuli from among the internal and external inputs (Palaniyappan and Liddle, 2012). Overall, interactions among these networks are thought to underlie nearly all cognitive functions (Menon, 2011). Research investigating functional connectivity during ASMR-trigger video viewing has reported that increased connectivity within the DMN is associated with mentalization, while increased connectivity centered on the anterior cingulate cortex—a key component of the SN—is linked to mental and physical self-referential processing (Lee et al., 2020).

Although changes in functional connectivity associated with ASMR have been reported, no studies to date have directly compared these changes with those induced by other auditory stimuli. Therefore, the present study aimed to compare functional connectivity changes during exposure to ASMR triggers and a control auditory stimulus (classical music) and to identify connectivity changes that are more strongly elicited by ASMR triggers. Previous studies have shown that listening to classical music is associated with autobiographical memory recall and self-referential processing, whereas ASMR has been linked to increased DMN connectivity and self-related processing centered on the anterior cingulate cortex. Based on these findings, we hypothesized that although both auditory stimuli would involve DMN-related connectivity changes, ASMR triggers would elicit stronger changes in the salience network (SN), which is involved in somatosensory and self-related processing. This study aims to clarify how functional connectivity during ASMR listening differs from that elicited by other auditory stimuli, providing important insights into the diversity of neural responses to external stimuli.

## 2 Materials and methods

### 2.1 Participants

We recruited 48 healthy volunteers (25 males and 23 females; age 20.7 ± 1.2 years). When deciding the sample size for this study, we referred to a previous fMRI study comparing brain activity during ASMR and classical music and selected a larger sample size than that used in the study (i.e., 30) (Sakurai et al., 2021). Individuals with a history of psychiatric disorders or any contraindications to MRI were excluded. This study did not collect information on participants’ musical training or musical experience.

The study protocol was approved by the Ethics Committee of Niigata University of Health and Welfare (approval No. 19281-240,514) and conducted in accordance with the Declaration of Helsinki. Written informed consent was obtained from all participants after they were informed of the study’s purpose, procedures, safety considerations, and potential risks.

### 2.2 Stimuli and task

ASMR triggers are commonly grouped into categories such as visual, tactile, repetitive sounds, simulation, and mouth sounds. Auditory-only triggers were selected for this study because auditory input is a primary driver of ASMR and is generally more effective than visual triggers (Barratt et al., 2017). For the ASMR trigger, we used whispering voices from publicly available online videos. Among these, mouth sounds readily elicit ASMR, particularly whispering, readily induce tingling sensations, and were therefore chosen for the ASMR condition (Barratt and Davis, 2015). For this study, the ASMR trigger consisted of Japanese audio selected to match the participants’ first language; the spoken content was composed primarily of onomatopoeia, thereby minimizing the influence of linguistic meaning as much as possible.

The comparison stimulus was the first movement of Ludwig van Beethoven’s Piano Sonata No. 14. This piece is notated in C-sharp minor, marked *Adagio sostenuto*, and in 2/2 time. Previous studies have reported that this piece is associated with a sense of calm and decreasing heart rate (Darki et al., 2022). In the present study, we selected this piece not as a form of classical music chosen to match the acoustic characteristics of ASMR triggers, but rather as a non-ASMR stimulus that has been reported to be associated with calming responses similar to those elicited by ASMR triggers. Therefore, it should not be interpreted as a representative or standard classical music stimulus. While the ASMR stimulus was an audio-based stimulus that included whispering, the comparison stimulus was a nonverbal musical piece produced by a single instrument; thus, the two stimuli differed in terms of stimulus format and structural organization. To characterize the acoustic properties of the stimuli, a frequency analysis was performed using Audacity version 3.7.7. Both the ASMR and classical music stimuli had a sampling frequency of 48 kHz and a bit depth of 16 bits. For the ASMR stimulus, the maximum peak was observed at 47 Hz, with multiple peaks detected between 300 and 6,000 Hz. In contrast, the classical music stimulus exhibited a maximum peak at approximately 411 Hz, with frequency components distributed across approximately 100–8,000 Hz. However, sound pressure levels were not measured, loudness consistency across conditions was not controlled, and amplitude normalization was not performed.

Each participant underwent three experimental conditions (rest, ASMR, and classical music). Following the acquisition of structural images, the rest condition was always administered first, whereas the order of the ASMR and classical music conditions was randomized across participants. Each condition was administered once, with a duration of 5 min per session. During each session, participants either listened continuously to the auditory stimulus or maintained a state of rest without any auditory stimulus. Auditory stimuli were delivered using MRI-compatible headphones with high noise isolation (iMag, Star Product, Tokyo, Japan).

### 2.3 MRI acquisition

MRI data were acquired on a 3 T Vantage Galan scanner (Canon Medical Systems, Tochigi, Japan) with a 32-channel head coil. T1-weighted structural images were obtained using a magnetization-prepared rapid gradient-echo (MP-RAGE) sequence with the following parameters: repetition time (TR) = 5.8 ms, echo time (TE) = 2.7 ms, TI = 900 ms, flip angle = 9°, matrix = 256 × 256, field of view (FOV) = 230 × 230 mm, and slice thickness = 1.2 mm. Following the structural scan, fMRI data were acquired while the participants rested with their eyes open. Functional images were obtained using an echo planar imaging (EPI) sequence with TR = 2000 ms (2.0 s), TE = 25 ms, flip angle = 85°, matrix = 64 × 64, FOV = 240 × 240 mm, and slice thickness = 3 mm. The scanning room was kept dark while data were collected.

### 2.4 Questionnaire

After completing the MRI scan, participants completed a questionnaire regarding somatosensory experiences during fMRI acquisition while listening to ASMR trigger sounds and classical music. The participants were asked whether they experienced a tingling sensation extending from the top of the head to the back while listening to ASMR triggers and classical music. Responses were rated on a 5-point Likert scale: 1. Strongly felt, 2. Felt, 3. Neither, 4. Did not feel much, and 5. Did not feel it.

Statistical analysis was performed using IBM SPSS Statistics version 27 (IBM Corporation, Armonk, NY, USA). To examine the difference in rating values between the ASMR-trigger and classical-music conditions, the Wilcoxon signed-rank test was performed. A two-tailed test was conducted, and the significance level was set at *α* = 0.05.

### 2.5 Data analysis

Preprocessing and analyses were performed using Statistical Parametric Mapping (SPM12) and the CONN functional connectivity toolbox (v17) implemented in MATLAB (MathWorks, Natick, MA, USA). Functional connectivity analyses proceeded in four steps: spatial preprocessing, temporal preprocessing, first-level (subject-level) analysis, and second-level (group-level) analysis.

Spatial preprocessing included slice-timing correction, motion correction, spatial normalization, and spatial smoothing. We used the ART-based functional outlier detection implemented in the CONN toolbox to detect body movements. We excluded scans as outliers if the variation in the global signal exceeded 3 standard deviations or if head movement exceeded 0.5 mm. For spatial standardization, the data were standardized to the Montreal Neurological Institute (MNI) standard brain, which is compliant with the International Consortium for Brain Mapping (ICBM) 152, to account for individual differences in brain shape. For spatial smoothing, a 3D Gaussian filter with a half-width of 8 mm was applied to mitigate image noise and individual brain variability resulting from the processing steps. For time-series preprocessing, we regressed scrubbing regressors—which included white matter, CSF, head motion parameters, and outlier scans—as confounding factors and then applied a bandpass filter with a bandwidth of 0.008–0.09 Hz.

For the individual analysis, we performed a region of interest (ROI)-to-ROI analysis. Time-series data for each ROI were extracted as values representative of the BOLD signal time series within the ROI. Table 1 shows the brain regions used for the ROI and their MNI coordinates. The analyzed ROIs were defined based on the standard ROI templates included in the CONN toolbox. We calculated the correlation coefficients for the time-series data between each ROI and applied Fisher’s z-transformation to the resulting Pearson correlation coefficients. The networks analyzed were the DMN, SN, FPN, dorsal attention network, visual network, language network, sensorimotor network, and cerebellar network included in the CONN toolbox.

**Table 1:** Brain regions and MNI coordinates used for the ROI.

Network	Brain region	ROI		
		*X*	*Y*	*Z*
Default mode network	Medial prefrontal cortex	1	55	−3
	Lateral parietal cortex (L)	−39	−77	33
	Lateral parietal cortex (R)	47	−67	29
	Posterior cingulate cortex	1	−61	38
Frontoparietal network	Lateral prefrontal cortex (L)	−43	33	28
	Posterior parietal cortex (L)	−46	−58	49
	Lateral prefrontal cortex (R)	41	38	30
	Posterior parietal cortex (R)	52	−52	45
Salience network	Anterior cingulate cortex	0	22	35
	Anterior insula (L)	−44	13	1
	Anterior insula (R)	47	14	0
	Rostral prefrontal cortex (L)	−35	45	27
	Rostral prefrontal cortex (R)	32	46	27
	Supramarginal gyrus (L)	−60	−39	31
	Supramarginal gyrus (R)	62	−35	32
Visual network	Visual.Medial	2	−79	12
	Visual.Occipital	0	−93-	−4
	Visual.Lateral (L)	−37	−79	10
	Visual.Lateral (R)	38	−72	13
Sensorimotor network	SensoriMotor.Lateral (L)	−55	−12	29
	SensoriMotor.Lateral (R)	56	−10	29
	SensoriMotor.Superior	0	−31	67
Dorsal attention network	Frontal Eye Field (L)	−27	−9	64
	Frontal Eye Field (R)	30	−6	64
	Intraparietal Sulcus (L)	−39	−42	54
	Intraparietal Sulcus (R)	39	−42	54
Language network	Inferior Frontal Gyrus (L)	−51	26	2
	Inferior Frontal Gyrus (R)	54	28	1
	Posterior Superior Temporal Gyrus (L)	−57	−47	15
	Posterior Superior Temporal Gyrus (R)	59	−42	13
Cerebellar network	Cerebellar.Anterior	0	−63	−30
	Cerebellar.Posterior	0	79	−32

In the group analysis, we compared the ASMR condition with the resting condition and the classical-music condition with the resting condition to evaluate changes in functional connectivity under each condition. The purpose of these comparisons was to demonstrate the changes in functional connectivity induced by each auditory stimulus relative to the resting state. We also directly compared the ASMR and classical-music conditions. This comparison evaluates the relative differences in coupling between two types of complex auditory stimuli. A one-sample t-test was performed at the group level for each contrast. The false discovery rate (FDR) was used for multiple comparison correction, and the significance level was set at *p* < 0.05.

To examine the relationship between subjective ratings and functional connectivity under ASMR conditions, we assessed the correlation between the *Z*-scores that represented functional connectivity and showed significant differences under the ASMR condition and the 5-point Likert scale for ASMR using Spearman’s rank correlation coefficient. The *Z*-score is the *Z*-transformed value of the Pearson correlation coefficient calculated from the synchronized BOLD signal time-series data between the two ROIs. The correlation coefficients were calculated using IBM SPSS Statistics Version 27. The significance level was set at *p* < 0.05.

Furthermore, to account for individual differences in ASMR responsiveness, we conducted additional analyses to verify whether the major functional connectivity differences identified in the main analysis were reproducible among participants who experienced ASMR. Participants who responded “1. Felt it very strongly” or “2. Felt it” in the subjective assessment during ASMR trigger listening were classified as “responders” (*n* = 42). Analyses were then repeated exclusively within this subgroup, focusing on the connectivity strength of the ROI pairs that showed significance in the main analysis. Specifically, paired *t*-tests were conducted for the single ROI pair that was significant in the ASMR condition and for the three ROI pairs that were significant in the direct comparison between the ASMR and classical music conditions. FDR correction was used for multiple comparison correction.

## 3 Results

### 3.1 Questionnaire

Table 2 shows the subjective ratings of somatosensory experiences elicited by ASMR triggers and classical music listening. Subjective tingling sensations were significantly stronger during ASMR listening than during classical music listening (*p* < 0.001).

**Table 2:** Subjective evaluation of somatosensory experiences during ASMR triggers and classical music listening.

Condition	1. Strongly felt	2. Felt	3. Neither	4. Did not feel much	5. Did not feel it	Average (SD)	*p*-value
ASMR trigger	18 (37.5%)	24 (50%)	1 (2.08%)	4 (8.33%)	1 (2.08%)	1.87 (0.940)	<0.001
Classical music	0 (0%)	6 (12.5%)	7 (14.6%)	13 (27.1%)	(22) (45.8%)	4.08 (1.05)	

Results of evaluating tingling sensation intensity using a 5-point Likert scale (1. Strongly felt, 2. Felt, 3. Neither, 4. Did not feel much, and 5. Did not feel) are shown. Tingling sensations were significantly stronger during ASMR listening than during classical music listening. Statistical analysis was performed using the Wilcoxon signed-rank test. A two-tailed test was conducted at a significance level of 0.05%. ASMR, autonomous sensory meridian response; SD, standard deviation.

### 3.2 fMRI

**Figure 1:**
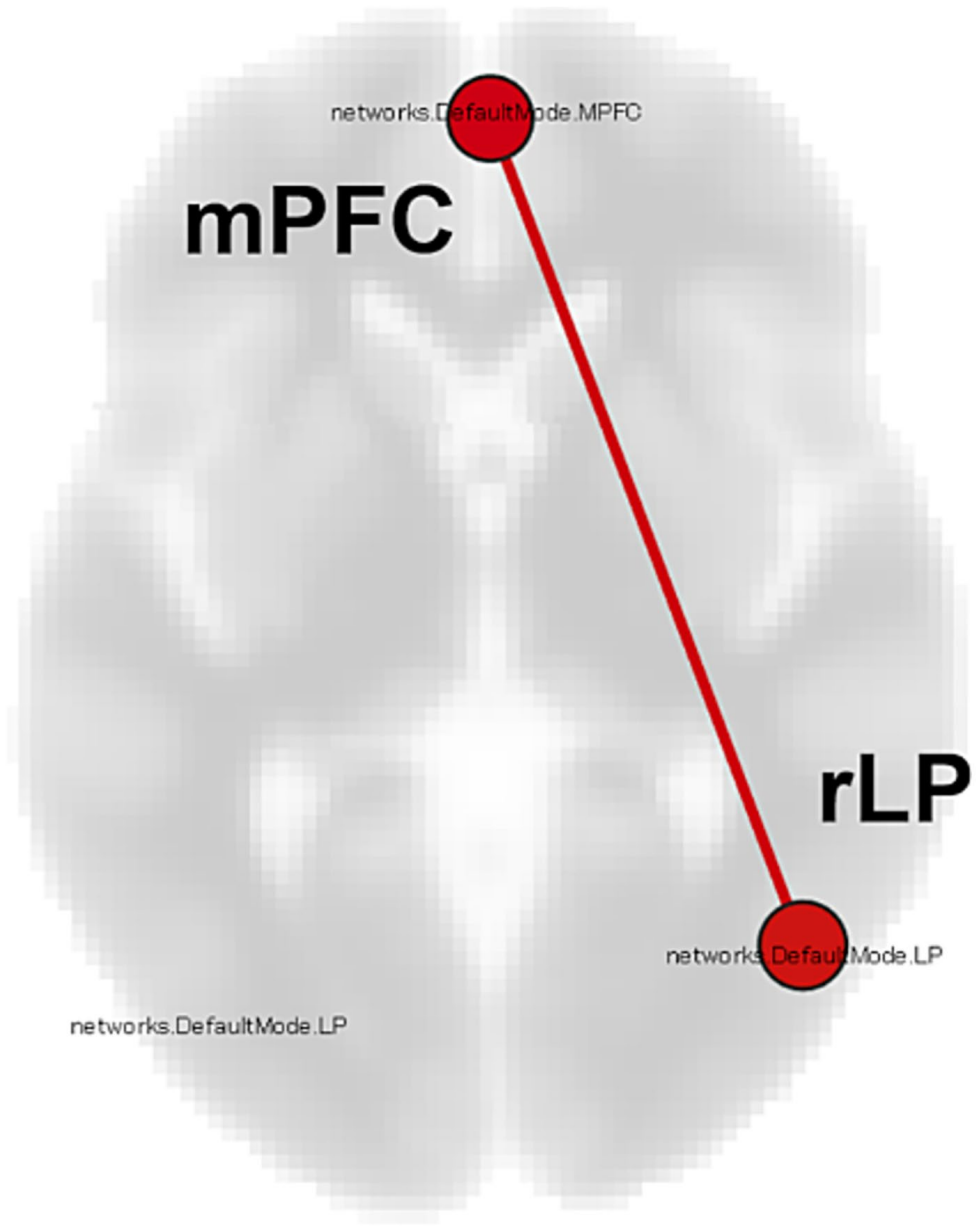
Brain regions showing changes in functional connectivity when listening to ASMR triggers. The image shows the brain regions with a significant difference in connectivity observed when resting-state connectivity was subtracted from the functional connectivity observed during ASMR trigger exposure. Warm-colored lines indicate higher functional connectivity during ASMR trigger exposure compared to that during the resting state. Connectivity between the medial prefrontal cortex and right lateral parietal cortex was increased. LP; lateral parietal cortex, mPFC; medial prefrontal cortex.

Figure 1, Table 3 show the brain regions exhibiting changes in functional connectivity during exposure to ASMR triggers. The triggers increased connectivity within the DMN between the medial prefrontal cortex (mPFC) and right lateral parietal cortex (LP).

**Table 3:** Brain regions showing changes in functional connectivity while listening to ASMR triggers.

Brain region		*T* value	*p*-value
Medial prefrontal cortex	Right lateral parietal cortex	12.59	<0.001

ASMR, autonomous sensory meridian response; MNI, Montreal Neurological Institute and Hospital.

**Figure 2:**
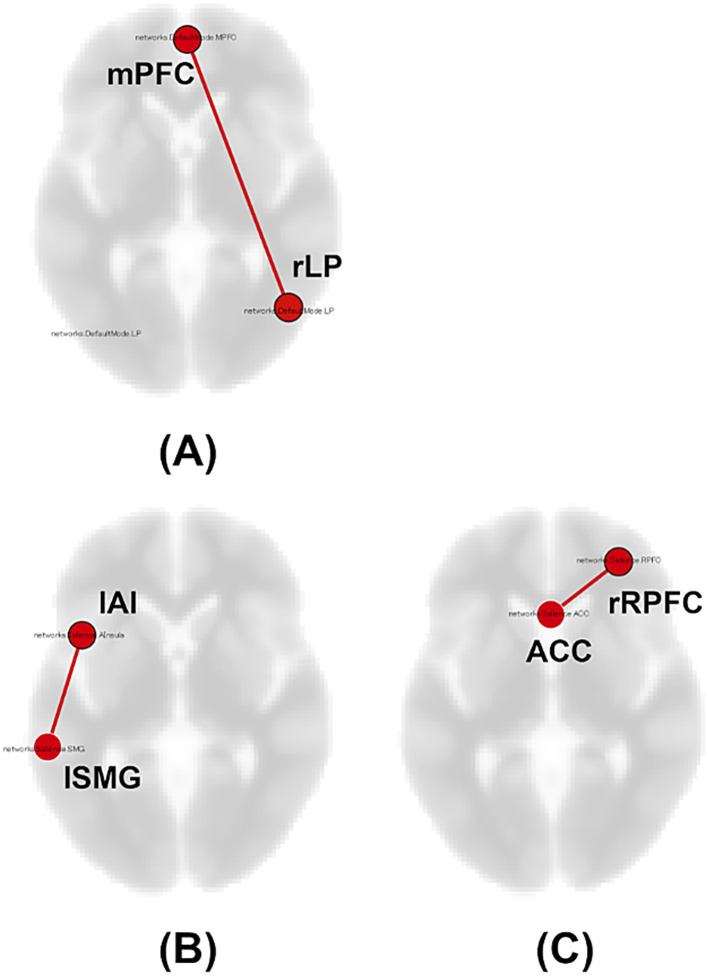
Difference between neural activity when listening to an ASMR trigger and when listening to classical music. The images show the brain regions for which significant differences were observed when functional connectivity during ASMR trigger listening was subtracted from that during classical music listening. Warm-colored lines indicate higher functional connectivity during ASMR trigger listening compared to classical music listening. The connectivities between the **(A)** right lateral parietal cortex and medial prefrontal cortex, **(B)** left insula and left supramarginal gyrus, **(C)** right rostral prefrontal cortex and anterior cingulate cortex were increased. mPFC; medial prefrontal cortex, LP lateral parietal cortex, AI anterior insula, SMG supramarginal gyrus, rRPFC rostral prefrontal cortex, ACC anterior cingulate cortex.

Figure 2, Table 4 show the results of subtracting the functional connectivity observed while listening to classical music from that observed while listening to ASMR triggers. When comparing the functional connectivity observed during ASMR trigger listening to that observed during classical music listening, ASMR triggers were associated with increased connectivity within the DMN, specifically in the medial prefrontal cortex and right lateral parietal cortex, as well as increased connectivity in the SN, specifically in the left anterior insular cortex, left supramarginal gyrus, right rostral prefrontal cortex, and anterior cingulate cortex.

**Table 4:** ASMR–classical music contrast in functional connectivity.

Brain region		*T* value	*p*-value
Medial prefrontal cortex	Right lateral parietal cortex	1.92	0.040
Left anterior insula	Left supramarginal gyrus	2.95	0.017
Right rostral prefrontal cortex	Anterior cingulate cortex	3.71	0.001

ASMR, autonomous sensory meridian response.

On the other hand, no significant changes in functional connectivity were observed when listening to classical music.

### 3.3 Correlation analysis

**Figure 3:**
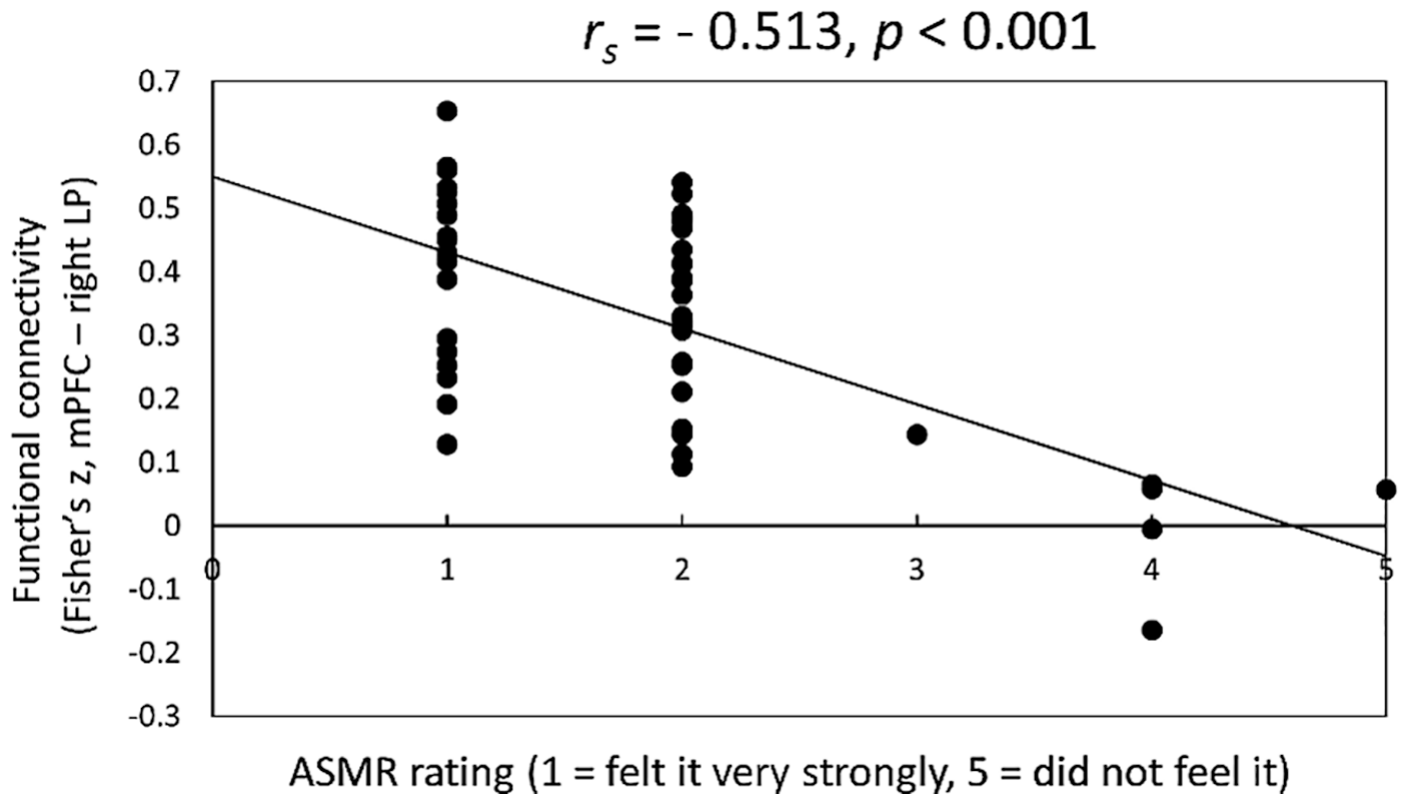
Relationship between ASMR intensity and functional connectivity between the medial prefrontal cortex and the right lateral parietal cortex during the ASMR condition. The correlation between the *Z*-scores for brain regions where functional connectivity changed significantly under ASMR conditions and ratings of somatosensory sensations in response to ASMR triggers during fMRI scanning was analyzed. A significant negative correlation was observed between functional connectivity strength between the medial prefrontal cortex and the right lateral parietal cortex and the intensity of somatosensory sensations in response to ASMR triggers during fMRI scanning (Spearman’s *rs* = −0.513, *p* < 0.001). The assessment of the intensity of these somatosensory sensations was conducted using a 5-point Likert scale: 1. Felt it very strongly, 2. Felt it, 3. Neither, 4. Did not feel it very much, 5. Did not feel it.

Figure 3 shows the association between the intensity of ASMR sensations and functional connectivity under ASMR conditions. A significant negative correlation was observed between ASMR ratings and the *Z* values representing functional connectivity between the medial prefrontal cortex and the right lateral parietal cortex (Spearman’s *rs =* −0.513*, p* < 0.001).

### 3.4 Analysis of ASMR responders

**Figure 4:**
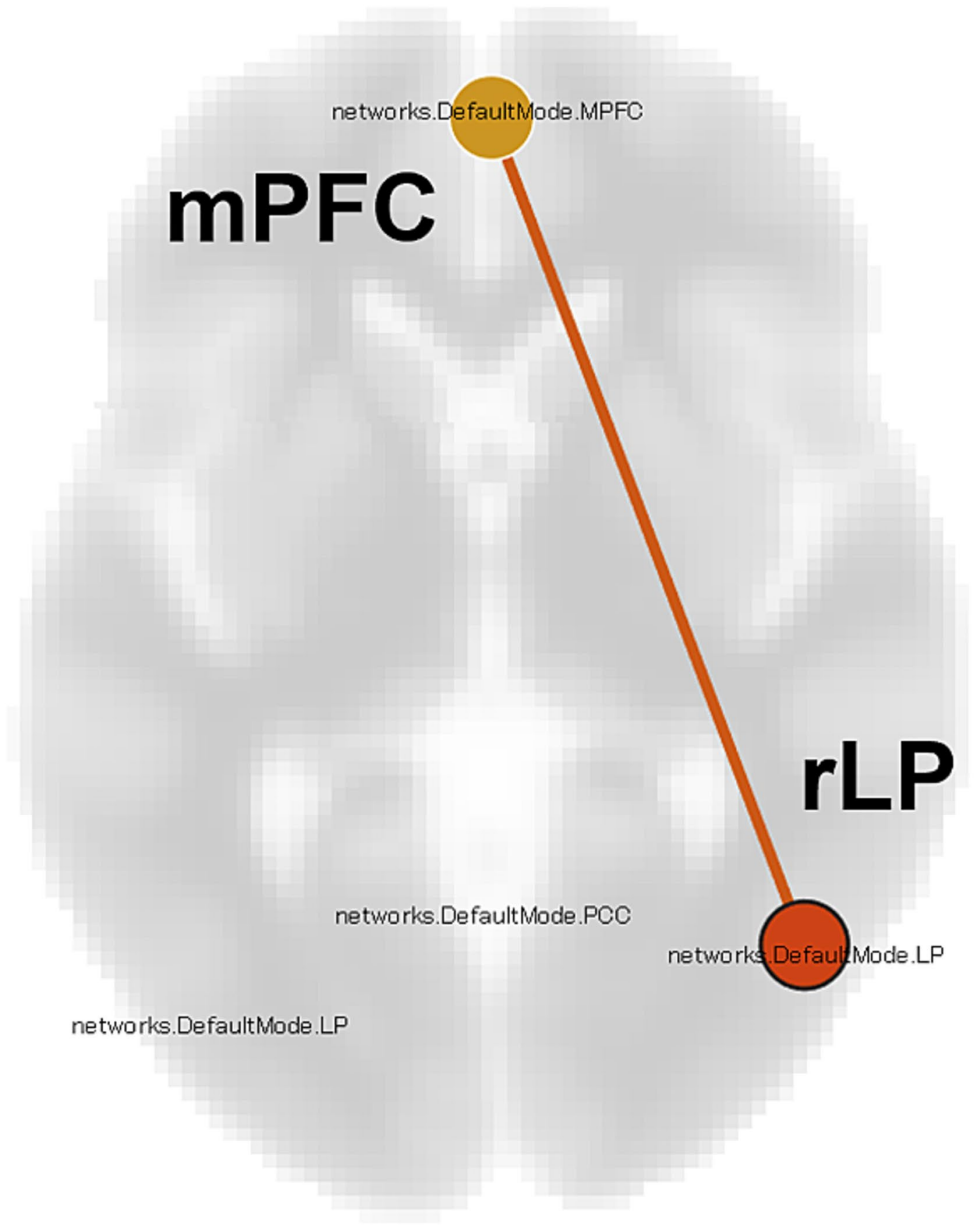
Brain regions showing changes in functional connectivity during ASMR trigger listening in ASMR responders. The image shows the brain regions with a significant difference in connectivity observed when ASMR responders resting-state connectivity was subtracted from the functional connectivity observed during ASMR trigger exposure. Warm-colored lines indicate higher functional connectivity during ASMR trigger exposure compared to that during the resting state. Connectivity between the medial prefrontal cortex and right lateral parietal cortex was increased. LP; lateral parietal cortex, mPFC; medial prefrontal cortex.

**Figure 5:**
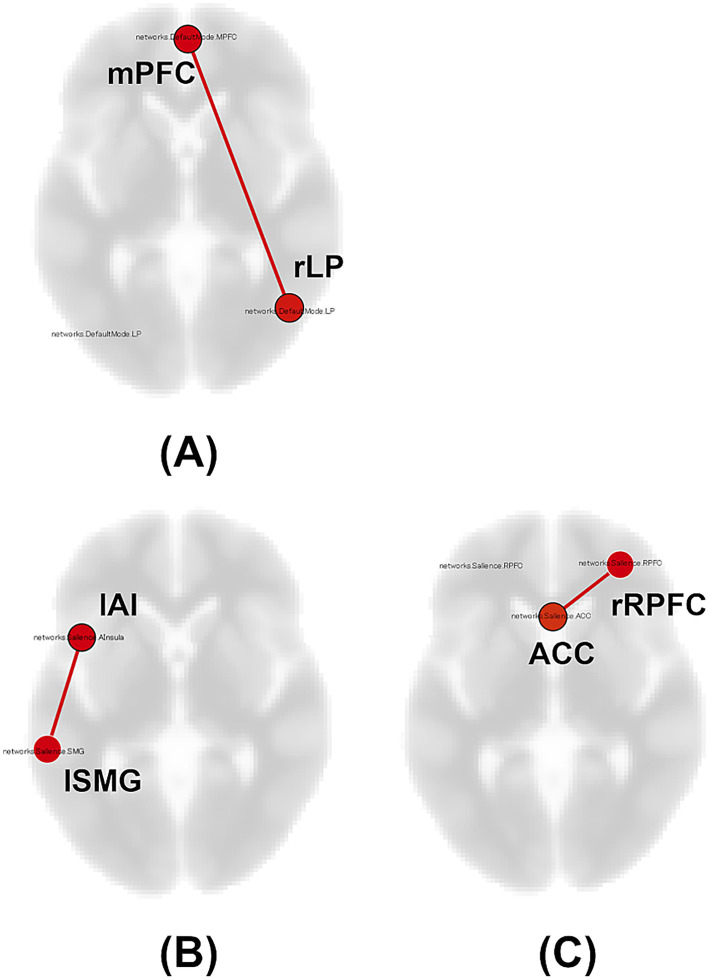
Differences in functional connectivity between ASMR trigger listening and classical music listening in ASMR responders. The images show the brain regions for which significant differences were observed when functional connectivity during ASMR trigger listening was subtracted from that during classical music listening. Warm-colored lines indicate higher functional connectivity during ASMR trigger listening compared to classical music listening. The connectivities between the **(A)** right lateral parietal cortex and medial prefrontal cortex, **(B)** left insula and left supramarginal gyrus, **(C)** right rostral prefrontal cortex and anterior cingulate cortex were increased. mPFC: medial prefrontal cortex, LP; lateral parietal cortex, AI; anterior insula, SMG; supramarginal gyrus, rRPFC; rostral prefrontal cortex, ACC; anterior cingulate cortex.

An additional analysis was conducted in responders. Figure 4, Table 5 show the brain regions showing changes in functional connectivity during ASMR trigger listening in responders. Figure 5, Table 6 present the results of the direct comparison between functional connectivity during ASMR trigger listening and classical music listening in this subgroup. During ASMR triggers, responders showed increased connectivity between the medial prefrontal cortex and the right lateral parietal cortex within the DMN, consistent with the findings of the main analysis. Furthermore, compared with classical music listening, ASMR triggers increased connectivity between the medial prefrontal cortex and the right lateral parietal cortex within the DMN, as well as between the left anterior insular cortex and the left supramarginal gyrus, and between the right rostral prefrontal cortex and the anterior cingulate cortex within the SN. These findings were also consistent with the main analysis.

**Table 5:** Brain regions showing changes in functional connectivity while listening to ASMR triggers.

Brain region		*T* value	*p*-value
Medial prefrontal cortex	Right lateral parietal cortex	18.18	<0.001

ASMR, autonomous sensory meridian response.

**Table 6:** ASMR–classical music contrast in functional connectivity.

Brain region		*T* value	*p*-value
Medial prefrontal cortex	Right lateral parietal cortex	2.12	0.031
Left anterior insula	Left supramarginal gyrus	3.42	0.021
Right rostral prefrontal cortex	Anterior cingulate cortex	4.11	0.002

ASMR, autonomous sensory meridian response.

## 4 Discussion

In this study, we compared functional connectivity patterns during listening to ASMR trigger sounds and the first movement of Piano Sonata No. 14, which served as a non-ASMR auditory stimulus. The results showed that, under the ASMR condition, connectivity between certain ROIs within the DMN and SN was higher than that observed under the comparison stimulus condition. These findings suggest that listening to ASMR trigger sounds may be associated with stronger network interactions related to self-related and internally evaluative processing, in addition to sensory processing, than listening to the comparison stimulus.

When listening to ASMR triggers, connectivity increased between the medial prefrontal cortex and right lateral parietal cortex within the DMN. The medial prefrontal cortex regulates emotions and evaluates self-relevant situations, and functional connectivity involving the medial prefrontal cortex is deeply involved in self-referential processing (Molnar-Szakacs and Uddin, 2013; Xu et al., 2019). Moreover, the lateral parietal cortex is involved in episodic memory and theory of mind, and the right lateral parietal cortex identifies and processes the type and intensity of emotions (Humphreys and Lambon Ralph, 2015; Lettieri et al., 2019). Tajik-Parvinchi et al. (2020) reported that increased connectivity between the medial prefrontal cortex and lateral parietal cortex is associated with emotional regulation. Therefore, the increased connectivity between these brain regions in this study suggests that self-related or internally evaluative processing was relatively heightened when listening to ASMR. In this regard, Morales et al. (2021) reported that ASMR is not merely a sensory response but is closely linked to emotions. In addition, participants who reported stronger ASMR sensations tended to exhibit stronger functional connectivity between the medial prefrontal cortex and the right lateral parietal cortex during ASMR listening. This finding suggests that individual differences in subjective ASMR intensity may be associated with the strength of connectivity between these regions. However, these results are correlational in nature and should not be interpreted as evidence of a causal relationship.

In a direct comparison between ASMR triggers and classical music, increased connectivity was observed between the medial prefrontal cortex and right lateral parietal cortex within the DMN, between the left anterior insular cortex and left supramarginal gyrus within the SN, and between the right rostral prefrontal cortex and anterior cingulate cortex. These findings indicate differences in relative functional connectivity between the two types of auditory stimuli. Increased connectivity between the medial prefrontal cortex and right lateral parietal cortex was also indicated by the analyses of individual participants who listened to ASMR triggers. This suggests that listening to ASMR triggers may be associated with stronger self-related evaluation and internally oriented processing than listening to classical music.

Furthermore, the connectivity between the left anterior insular cortex and left supramarginal gyrus, as well as between the right rostral prefrontal cortex and anterior cingulate cortex, increased more when listening to ASMR triggers than when listening to classical music. Previous studies have reported that connectivity between the insular cortex and supramarginal gyrus may be associated with DMN-related activity and reflective thinking (Andrews-Hanna et al., 2014; Chen et al., 2025; Satyshur et al., 2018). Furthermore, ASMR has been associated with increased alpha waves, which are linked to self-referential processing, as well as increased DMN connectivity (Lee et al., 2020; Swart et al., 2022). Given these findings, the increased connectivity between the left anterior insular cortex and left superior marginal gyrus observed in this study suggests that ASMR triggers may involve networks more closely associated with internal information processing, compared with classical music. Moreover, the anterior cingulate cortex is involved in higher-order sensory integration, including both interoceptive and exteroceptive sensations (Sasaoka et al., 2024), while the rostral prefrontal cortex is involved in the perception of environmental stimuli and the evaluation of interoceptive sensations (Burt et al., 2023). Ueno et al. reported that the connection between the anterior cingulate cortex and rostral prefrontal cortex is associated with the maintenance of interoceptive sensations (Ueno et al., 2020). These findings suggest that listening to ASMR triggers may be associated with stronger sensory integration involving internal processing and bodily sensations than listening to control stimuli. This interpretation is consistent with the results of the present study, in which subjective ratings of somatosensory sensations were significantly stronger for ASMR triggers than for control stimuli. Furthermore, similar major changes in connectivity were observed in an additional analysis limited to participants who subjectively experienced ASMR. This suggests that the interpretation of the connectivity differences demonstrated in this study may also be applicable to participants who reported subjective ASMR.

No significant changes in functional connectivity were observed in the analysis of classical music alone. A possible reason for this is a single piano piece was used as the comparison stimulus and subjective emotional responses to the musical stimuli were not assessed; consequently, individual differences in responses to the stimuli may not have manifested as consistent connectivity changes at the group level.

The statistically significant differences observed in this study were limited to a small number of ROI pairs. Therefore, it is appropriate to interpret the findings of this study as differences localized to specific inter-ROI connections rather than as widespread changes across the entire brain network. This study has some limitations. First, no preliminary screening for ASMR sensitivity was conducted. Therefore, the results of this study should be interpreted as averages for a population that includes participants with varying levels of ASMR responsiveness. Second, since we did not measure sound pressure levels, perform loudness matching between conditions, or normalize amplitudes between the ASMR and classical music stimuli, the observed differences between conditions may have been influenced not only by the stimuli content but also by their acoustic properties. Therefore, the differences in functional connectivity observed between conditions in this study do not directly indicate ASMR-specific neural mechanisms, but should be interpreted as relative differences between two specific types of auditory stimuli. Third, since only one type each of ASMR trigger and classical music were used in this study, stimulus-specific structural characteristics—such as the speaker’s voice quality or the melodic structure of the music—may have influenced the results. Furthermore, the classical-music condition utilized a single musical excerpt: the first movement of Beethoven’s Piano Sonata No. 14. Therefore, the findings of this study should be interpreted not as a comparison with classical music in general, but as a contrast between ASMR stimuli and a specific comparison stimulus. In addition, because information on participants’ musical training and musical experience was not collected, it cannot be ruled out that individual differences in musical experience may have influenced subjective and neural responses to classical music. In future studies, presenting ASMR triggers from multiple categories or stimuli tailored to participants’ preferences will allow for a more detailed investigation of the neural basis of ASMR. Fourth, this study did not systematically record feelings of pleasure or emotional changes that arose when listening to the stimulus; consequently, it is not possible to sufficiently distinguish whether the observed differences in functional connectivity reflect ASMR-specific processing or differences in pleasure, displeasure, or emotional responses associated with the stimuli. Fifth, since the subjective assessments were based on a 5-point Likert scale administered after the scan and showed a skewed distribution, the association between subjective assessments and functional connectivity should be considered an exploratory finding. Furthermore, the questionnaire included an item regarding a tingling sensation extending from the top of the head to the back; however, the origin and spread of the sensation were not assessed as separate items. Therefore, the subjective ratings in this study should be interpreted as reflecting the intensity of this tingling sensation rather than as a detailed confirmation of the bodily distribution pattern typically associated with ASMR. Sixth, because the participants in this study were limited to healthy young adults, the generalizability of the results is limited.

## 5 Conclusion

Using fMRI, this study compared functional connectivity while participants listened to ASMR triggers and classical music to examine ASMR-specific brain activity. When listening to ASMR triggers, the connectivity within the DMN and SN increased more than when listening to classical music. These findings suggest that, during exposure to ASMR triggers, in addition to self-related processing, information processing related to sensory integration involving bodily sensations may be more involved compared with listening to the control stimulus.

## Data Availability

The raw data supporting the conclusions of this article will be made available by the authors, without undue reservation.

## References

[ref1] Andrews-Hanna J. R. Smallwood J. Spreng R. N. (2014). The default network and self-generated thought: component processes, dynamic control, and clinical relevance. Ann. N. Y. Acad. Sci. 1316, 29–52. doi: 10.1111/nyas.12360, 24502540 PMC4039623

[ref2] Barratt E. L. Davis N. J. (2015). Autonomous sensory meridian response (ASMR): a flow-like mental state. PeerJ 3:e851. doi: 10.7717/peerj.851, 25834771 PMC4380153

[ref3] Barratt E. L. Spence C. Davis N. J. (2017). Sensory determinants of the autonomous sensory meridian response (ASMR): understanding the triggers. PeerJ 5:e3846. doi: 10.7717/peerj.3846, 29018601 PMC5633022

[ref4] Blood A. J. Zatorre R. J. (2001). Intensely pleasurable responses to music correlate with activity in brain regions implicated in reward and emotion. Proc. Natl. Acad. Sci. USA 98, 11818–11823. doi: 10.1073/pnas.191355898, 11573015 PMC58814

[ref5] Buckner R. L. Andrews-Hanna J. R. Schacter D. L. (2008). The brain’s default network: anatomy, function, and relevance to disease. Ann. N. Y. Acad. Sci. 1124, 1–38. doi: 10.1196/annals.1440.011, 18400922

[ref6] Burt J. S. Davenport M. P. Welch J. F. Davenport P. W. (2023). fNIRS analysis of rostral prefrontal cortex activity and perception of inspiratory loads. Respir. Physiol. Neurobiol. 316:104113. doi: 10.1016/j.resp.2023.104113, 37442516

[ref7] Chen Y. Li S. Li M. Huang L. Jiang M. Li G. . (2025). Aberrant static and dynamic functional connectivity of insular cortex in patients with trigeminal neuralgia. Sci. Rep. 15:21097. doi: 10.1038/s41598-025-07468-7, 40596598 PMC12217799

[ref8] Darki C. Riley J. Dadabhoy D. P. Darki A. Garetto J. (2022). The effect of classical music on heart rate, blood pressure, and mood. Cureus 14:e27348. doi: 10.7759/cureus.27348, 36046316 PMC9417331

[ref9] Humphreys G. F. Lambon Ralph M. A. (2015). Fusion and fission of cognitive functions in the human parietal cortex. Cereb. Cortex 25, 3547–3560. doi: 10.1093/cercor/bhu198, 25205661 PMC4585503

[ref10] Lee S. Kim J. Tak S. (2020). Effects of autonomous sensory meridian response on the functional connectivity as measured by functional magnetic resonance imaging. Front. Behav. Neurosci. 14:154. doi: 10.3389/fnbeh.2020.00154, 33192358 PMC7481390

[ref11] Lettieri G. Handjaras G. Ricciardi E. Leo A. Papale P. Betta M. . (2019). Emotionotopy in the human right temporo-parietal cortex. Nat. Commun. 10:5568. doi: 10.1038/s41467-019-13599-z, 31804504 PMC6895053

[ref12] Li L. Di X. Zhang H. Huang G. Zhang L. Liang Z. . (2022). Characterization of whole-brain task-modulated functional connectivity in response to nociceptive pain: a multisensory comparison study. Hum. Brain Mapp. 43, 1061–1075. doi: 10.1002/hbm.25707, 34761468 PMC8764484

[ref13] Lochte B. C. Guillory S. A. Richard C. A. H. Kelley W. M. (2018). An fMRI investigation of the neural correlates underlying the autonomous sensory meridian response (ASMR). Bioimpacts 8, 295–304. doi: 10.15171/bi.2018.32, 30397584 PMC6209833

[ref14] Menon V. (2011). Large-scale brain networks and psychopathology: a unifying triple network model. Trends Cogn. Sci. 15, 483–506. doi: 10.1016/j.tics.2011.08.003, 21908230

[ref15] Mitterschiffthaler M. T. Fu C. H. Y. Dalton J. A. Andrew C. M. Williams S. C. R. (2007). A functional MRI study of happy and sad affective states induced by classical music. Hum. Brain Mapp. 28, 1150–1162. doi: 10.1002/hbm.20337, 17290372 PMC6871455

[ref16] Molnar-Szakacs I. Uddin L. Q. (2013). Self-processing and the default mode network: interactions with the mirror neuron system. Front. Hum. Neurosci. 7:571. doi: 10.3389/fnhum.2013.00571, 24062671 PMC3769892

[ref17] Morales R. Ramírez-Benavides D. Villena-Gonzalez M. (2021). Autonomous sensory meridian response self-reporters showed higher scores for cognitive reappraisal as an emotion regulation strategy. PeerJ 9:e11474. doi: 10.7717/peerj.11474, 34123591 PMC8164417

[ref18] Palaniyappan L. Liddle P. F. (2012). Does the salience network play a cardinal role in psychosis? An emerging hypothesis of insular dysfunction. J. Psychiatry Neurosci. 37, 17–27. doi: 10.1503/jpn.100176, 21693094 PMC3244495

[ref19] Poerio G. L. Blakey E. Hostler T. J. Veltri T. (2018). More than a feeling: autonomous sensory meridian response (ASMR) is characterized by reliable changes in affect and physiology. PLoS One 13:e0196645. doi: 10.1371/journal.pone.0196645, 29924796 PMC6010208

[ref20] Sakurai N. Ohno K. Kasai S. Nagasaka K. Onishi H. Kodama N. (2021). Induction of relaxation by autonomous sensory meridian response. Front. Behav. Neurosci. 15:761621. doi: 10.3389/fnbeh.2021.761621, 34916914 PMC8669134

[ref21] Sasaoka T. Hirose K. Maekawa T. Inui T. Yamawaki S. (2024). The anterior cingulate cortex is involved in intero-exteroceptive integration for spatial image transformation of the self-body. NeuroImage 293:120634. doi: 10.1016/j.neuroimage.2024.120634, 38705431

[ref22] Satyshur M. D. Layden E. A. Gowins J. R. Buchanan A. Gollan J. K. (2018). Functional connectivity of reflective and brooding rumination in depressed and healthy women. Cogn. Affect. Behav. Neurosci. 18, 884–901. doi: 10.3758/s13415-018-0611-7, 29949111

[ref23] Smith S. D. Fredborg B. K. Kornelsen J. (2019). A functional magnetic resonance imaging investigation of the autonomous sensory meridian response. PeerJ 7:e7122. doi: 10.7717/peerj.7122, 31275748 PMC6590446

[ref24] Song L. Ren Y. Wang K. Hou Y. Nie J. He X. (2023). Mapping the time-varying functional brain networks in response to naturalistic movie stimuli. Front. Neurosci. 17:1199150. doi: 10.3389/fnins.2023.1199150, 37397459 PMC10311647

[ref25] Swart T. R. Banissy M. J. Hein T. P. Bruña R. Pereda E. Bhattacharya J. (2022). ASMR amplifies low frequency and reduces high frequency oscillations. Cortex 149, 85–100. doi: 10.1016/j.cortex.2022.01.004, 35189396

[ref26] Tajik-Parvinchi D. Davis A. Roth S. Rosenbaum P. Hopmans S. N. Dudin A. . (2020). Functional connectivity and quality of life in young adults with cerebral palsy: a feasibility study. BMC Neurol. 20:388. doi: 10.1186/s12883-020-01950-7, 33096988 PMC7583292

[ref27] Taruffi L. Pehrs C. Skouras S. Koelsch S. (2017). Effects of sad and happy music on mind-wandering and the default mode network. Sci. Rep. 7:14396. doi: 10.1038/s41598-017-14849-0, 29089542 PMC5663956

[ref28] Ueno D. Matsuoka T. Kato Y. Ayani N. Maeda S. Takeda M. . (2020). Individual differences in interoceptive accuracy are correlated with salience network connectivity in older adults. Front. Aging Neurosci. 12:592002. doi: 10.3389/fnagi.2020.592002, 33335482 PMC7736179

[ref29] Xu P. Chen A. Li Y. Xing X. Lu H. (2019). Medial prefrontal cortex in neurological diseases. Physiol. Genomics 51, 432–442. doi: 10.1152/physiolgenomics.00006.2019, 31373533 PMC6766703

